# Role of inflammation and evidence for the use of colchicine in patients with acute coronary syndrome

**DOI:** 10.3389/fcvm.2024.1356023

**Published:** 2024-06-27

**Authors:** Juan Francisco Bulnes, Leticia González, Leonardo Velásquez, María Paz Orellana, Paula Muñoz Venturelli, Gonzalo Martínez

**Affiliations:** ^1^ División de Enfermedades Cardiovasculares, Pontificia Universidad Católica de Chile, Santiago, Chile; ^2^ Centro de Imágenes Biomédicas, Departamento de Radiología, Pontificia Universidad Católica de Chile, Santiago, Chile; ^3^ Centro de Estudios Clínicos, Instituto de Ciencias e Innovación en Medicina (ICIM), Facultad de Medicina Clínica Alemana, Universidad del Desarrollo, Santiago, Chile; ^4^ Heart Research Institute, Sydney, NSW, Australia

**Keywords:** colchicine, acute coronary syndrome, myocardial infarction, inflammasome, NETosis

## Abstract

Acute Coronary Syndrome (ACS) significantly contributes to cardiovascular death worldwide. ACS may arise from the disruption of an atherosclerotic plaque, ultimately leading to acute ischemia and myocardial infarction. In the pathogenesis of atherosclerosis, inflammation assumes a pivotal role, not solely in the initiation and complications of atherosclerotic plaque formation, but also in the myocardial response to ischemic insult. Acute inflammatory processes, coupled with time to reperfusion, orchestrate ischemic and reperfusion injuries, dictating infarct magnitude and acute left ventricular (LV) remodeling. Conversely, chronic inflammation, alongside neurohumoral activation, governs persistent LV remodeling. The interplay between chronic LV remodeling and recurrent ischemic episodes delineates the progression of the disease toward heart failure and cardiovascular death. Colchicine exerts anti-inflammatory properties affecting both the myocardium and atherosclerotic plaque by modulating the activity of monocyte/macrophages, neutrophils, and platelets. This modulation can potentially result in a more favorable LV remodeling and forestalls the recurrence of ACS. This narrative review aims to delineate the role of inflammation across the different phases of ACS pathophysiology and describe the mechanistic underpinnings of colchicine, exploring its purported role in modulating each of these stages.

## 1 Introduction

Ischemic heart disease (IHD) continues to be the leading cause of mortality worldwide, despite substantial advancements in diagnosis and treatment (1). Considering the prevailing trends of IHD risk factors, cardiovascular (CV) mortality is expected to rise through 2025 (2). Acute coronary syndrome (ACS) significantly contributes to CV death, accounting for 49.2% of the global registered deaths in 2019 (3), underscoring the importance of understanding the pathophysiology of the disease to significantly impact CV morbidity and mortality. Atherosclerotic ACS encompasses various phenotypes, including unstable angina (UA), ST-segment elevation myocardial infarction (STEMI), and non-ST segment elevation myocardial infarction (NSTEMI) (4). The common denominator among the diverse pathobiological manifestations of ACS is the obstruction of coronary artery blood flow, most commonly due to the rupture or erosion of a pre-existing atherosclerotic plaque and the subsequent thrombus formation (5).

For decades, atherosclerosis has been understood as a disease primarily driven by lipids, particularly emphasizing the role of low-density lipoprotein (LDL) cholesterol as a central factor in its pathogenesis. In fact, the introduction of potent cholesterol-lowering medications like statins, along with other major advancements in ACS treatment, has led to a significant reduction in morbidity and mortality associated with this condition. However, despite these achievements, a substantial residual risk remains, with recurrence rates persisting at approximately 20% after 3 years and up to 40% after 10 years (6, 7). As a result, there has been a shift in focus towards exploring alternative pathophysiological pathways. It is now recognized that this residual atherosclerotic risk encompasses three main components: (i) residual lipidic components—primarily lipoprotein(a) and triglyceride-rich lipoproteins—residual thrombosis; and, notably, inflammation (8–12).

While the concept of inflammation driving atherosclerosis has been present since the 19th century, significant advancements in understanding its multifaceted roles in disease pathogenesis have been achieved only recently (13). Evidence has accumulated, highlighting inflammation as a central process in atherosclerotic plaque formation (12, 14). Moreover, it has become evident that the transition of a silent atherosclerotic lesion into a plaque prone to erosion or rupture is heavily influenced by activated immune and inflammatory cells that destabilize the lesion, leading to thrombosis and ischemia (15, 16).

Inflammation is a central component of innate immunity and serves as a crucial mechanism for neutralizing harmful agents or facilitating tissue repair (14). However, failure to resolve an inflammatory response can lead to a persistent state of low-grade inflammation, commonly observed in conditions associated with increased risk of IHD, such as type 2 diabetes mellitus and non-alcoholic fatty liver disease (17). This persistent inflammation can be local or systemic, and both forms of inflammation might coexist and overlap within an individual, albeit with varying relevance to each individual patient. In fact, systemic inflammatory disorders, such as rheumatoid arthritis and systemic lupus erythematosus are associated with increased cardiovascular risk, independent of other traditional risk factors, underscoring the pivotal role of inflammation as a driver of IHD (18, 19).

Given the significance of inflammation in residual atherosclerotic risk, there has been a shift towards developing therapies that specifically target inflammation to further enhance outcomes in this high-risk population. The CANTOS trial pioneered the investigation of the inflammatory hypothesis in atherothrombosis (20). In this study, patients with a history of myocardial infarction (MI) and elevated levels of high-sensitivity C-reactive protein (hs-CRP) (≥2 mg/dl) were randomly assigned to receive either canakinumab [an interleukin-1β (IL-1β) monoclonal antibody] or placebo. Canakinumab demonstrated a 15% reduction in cardiovascular events, independent of aggressive cholesterol control. Despite these promising results, immunosuppression with canakinumab was associated with an increase in infection-related mortality, thus limiting its widespread adoption (20). Several other anti-inflammatory agents have been investigated, but most have proved to be ineffective or potentially harmful (21–23). However, colchicine, an anti-inflammatory medication that inhibits microtubule polymerization and interferes with various stages of the inflammatory process (24), emerges as an appealing alternative due to recent evidence of its efficacy, coupled with its cost-effectiveness and favorable safety profile.

This narrative review outlines the role of inflammation in the distinct stages of ACS pathophysiology. It subsequently provides a comprehensive description of the mechanism of action of colchicine and presents the evidence regarding its impact on each delineated stage of ACS pathophysiology. Additionally, this review explores recent advancements in alternative anti-inflammatory approaches and their effects.

## 2 Pathophysiology of acute coronary syndrome. Focus on inflammation

Acute coronary syndromes most commonly arise from atherosclerotic plaque disruption (i.e., rupture or erosion), leading to thrombus formation and coronary obstruction (25). Subsequent myocardial ischemia results in cardiomyocyte death and adaptive changes that determine cardiac remodeling (26–28). Inflammation not only plays a central role in the development and complications of atherosclerotic plaque but also in the myocardial response to ischemic injury (29). Here, we briefly review the pathophysiology of ACS, focusing on the role of inflammation in 3 distinctive stages: (i) atherosclerotic plaque build-up and disruption; (ii) acute myocardial injury, and (iii) chronic left ventricular (LV) remodeling.

### 2.1 Atherosclerotic plaque build-up and disruption

Atherosclerotic plaque development results from the accumulation of apolipoprotein B-containing lipoproteins in the vessel intima, followed by monocyte infiltration (14). Monocyte-derived macrophages fail to eliminate the modified lipoproteins and transform into highly inflammatory foam cells, releasing Tumor Necrosis Factor-α (TNF-α) and interleukin-1β (IL-1β), exacerbating endothelial dysfunction and perpetuating the inflammatory response (30). Macrophage dysfunction also manifests as a failed attempt to clear apoptotic cells within plaque (a process called efferocytosis), which results in cell debris accumulation and early plaque development. Recently, it has been shown that failed efferocytosis relate to an enhance expression of the transcription factor GATA-2, which in turn results in the dysregulation of key regulatory proteins (31). Advanced atherosclerotic plaques are characterized by a lipid-rich core consisting of foam cells, cell debris, and extracellular cholesterol, as well as a fibrous cap composed of extracellular matrix and smooth muscle cells. In the later stages of the disease, plaque disruption may occur, potentially leading to an ACS (12, 14, 25). Autopsy studies and intravascular imaging through optical coherence tomography (OCT) have recognized three plaque phenotypes in atherosclerotic ACS: plaque rupture, plaque erosion, and eruptive calcific nodule, with the latter being significantly less common. The composition of the advanced plaque will determine whether it is prone to rupture or erosion (32, 33).

Ruptured plaques typically encompass a thin cap fibroatheroma (TCFA) overlying a large lipidic and necrotic core; when rupture occurs, the core components become exposed to the circulation, causing *in situ* thrombosis and impaired distal flow (16, 33). Inflammation has a key role in the pathophysiology of plaque rupture, with an abundance of macrophages and foam cells—along with, to a lesser extent, *T* cells- typically near the margins of the fissure (34, 35). Conversely, in eroded plaques, the fibrous cap remains intact, but erosion of the endothelial layer exposes the extracellular matrix (ECM) components to the circulation, initiating thrombosis (36). Unlike ruptured plaques, this phenotype typically involves fibrous plaques rich in ECM components -such as glycosaminoglycans and proteoglycans-, rather than lipids (37); and they have fewer inflammatory cells and more smooth vascular muscle cells (36). The pathophysiology of plaque erosion is complex and markedly distinct from that of ruptured plaques. This involves disturbances in local flow patterns and accumulation of subintimal low molecular weight hyaluronan, leading to the activation of toll-like receptor 2 (TLR 2) and the desquamation of endothelial cells (38–40). Subsequently, neutrophils react releasing neutrophil extracellular traps (NETs), as detailed below, with final platelet-rich thrombus generation (41, 42). Table 1 delineates the main characteristics distinguishing plaque rupture from plaque erosion.

**Table 1:** Histopathologic and pathophysiologic characteristics of plaque rupture vs. plaque erosion.

Plaque rupture	Plaque erosion
Larger lipidic/necrotic core	Smaller lipidic/necrotic core
Thin fibrous cap	Thick fibrous cap
Larger plaque burden	Smaller plaque burden
Inflammatory cell-rich (monocytes, macrophages and foam cells, T cells)	Smooth muscle cell rich Extracellular matrix-rich (Proteoglycan, glycosaminoglycan, low molecular weigh hyaluronan)
Monocyte predominance	Neutrophil predominance (with NETosis)
Red thrombus (platelet and fibrn-rich, with abundant red blood cells)	White thrombus (platelet rich, red blood cell-poor and NETs present)

As observed, macrophages play a pivotal role in the pathogenesis of TCFA and plaque rupture (43). In more advanced atherosclerotic lesions, macrophages accumulate within the plaque as cell debris and release matrix metalloproteinases, which degrade the fibrous cap, predisposing to plaque rupture (44, 45). The nucleotide-binding oligomerization domain-like receptor, pyrin domain-containing 3 (NLRP3) inflammasome is a multimeric protein complex responsible for generating IL-1β and interleukin 18 (IL-18) by monocyte/macrophages (18, 46). Elevated expression levels of NLRP3 inflammasome components have been detected in human atherosclerotic plaques (47), correlating with disease severity (48). Additionally, they have been found to be elevated in ACS patients compared to controls (49). Conversely, the administration of a specific NLRP3 inflammasome inhibitor—MCC950—results in less plaque development and a reduction in the inflammasome cytokine products in murine models (50, 51). Notably, peripheral monocytes in ACS patients appear to be “primed” for inflammasome activation (52), highlighting the importance of the IL-1β axis and proposing it as a potential target for therapeutic interventions, as demonstrated in the CANTOS trial (20).

Neutrophils play significant roles throughout all stages of atherosclerosis development (53). They promote foam cell formation and metalloproteinase-induced plaque instability (54, 55). They release extracellular matrix proteinases (i.e., elastase and proteinase-3) which contribute to plaque destabilization (56–59) and facilitate monocyte recruitment and activation (60, 61). Of particular relevance is their role in the pathogenesis of plaque erosion through NETosis. NETs are web-like structures composed of DNA, histones, neutrophil elastase, and myeloperoxidase, released upon neutrophil activation (62). This leads to neutrophil-platelet aggregation and cytokine release from macrophages, lymphocytes, and endothelial cells, creating a potent pro-inflammatory and pro-thrombotic environment (63–66). Furthermore, NETs have also been found in ruptured plaques (67) and can further stimulate macrophages for cytokine production, fostering the inflammatory milieu during an ACS (68, 69). On the other hand, inflammasome activation promotes neutrophil recruitment and NETs formation in atherosclerotic plaques (70). The key role of this NETs-monocyte cross-talk is further exemplified by a recent experiment by Schumski et al. (71). Here, the injection of lipopolysaccharide into hypercholesterolemic mice resulted in a myeloid-dependent increased of atherosclerotic plaque size and the deposition of NETs in the arterial lumen. Conversely, the inhibition of NETs release prevents lesion expansion secondary to lipopolysaccharide administration.

In addition to their role in thrombus formation, platelets have emerged as important regulators of inflammation and immune responses implicated in the onset and progression of atherosclerosis (72). They act as a key link between leukocytes and endothelial cells by binding to dysfunctional endothelium, thereby facilitating the recruitment of leukocytes to the subendothelial compartment and initiating the inflammatory cascade during atherogenesis (73). Moreover, platelets are increasingly recognized for their involvement in lipid metabolism, influencing monocyte differentiation into macrophages and modulating macrophage lipid accumulation, thus promoting foam cell formation and plaque destabilization (74). Additionally, platelets exhibit migratory capabilities, being attracted by cytokines and chemokines to migrate through endothelial barriers and actively translocate into atherosclerotic lesions, where they interact with monocytes and assist in their migration (75–77). Accordingly, studies in atherosclerotic-prone Ldlr^+/−^ mice have shown that induced platelet apoptosis via Bcl-x_L_ inhibition results in reduced atherosclerotic plaque formation (78).

The role of inflammation in the onset and progression of atherosclerosis is supported by the recognition of increased cardiovascular (CV) risk associated with the presence of clonal hematopoiesis of indeterminate potential (CHIP) (79). These age-acquired somatic mutations occurring in hematopoietic stem cells within the bone marrow may confer upon them a competitive advantage, thereby facilitating the accumulation of their progeny—including mutated macrophages and neutrophils—in the peripheral blood. The majority of CHIP cases can be attributed to mutations in only a handful of genes, with all but one of them implicated in DNA or histone methylation defects, potentially influencing the modulation of inflammatory gene expression (79). For instance, monocytes/macrophages carrying CHIP-related mutations in Tet2, DNMT3A, or JAK2VF promote inflammasome activation, thus exhibiting increased expression of IL-1 and IL-6, alongside other inflammatory mediators (79–81). On the other hand, neutrophils carrying the JAK2VF mutation exhibit heightened NETosis (82). In addition to supporting the role of inflammation in atherosclerosis, the CHIP hypothesis may serve as a plausible bridge connecting age and atherosclerosis, which may occur, at least partly, through inflammation (83).

Following an initial ACS patients remain at increased CV risk, experiencing recurrent events in up to 20% of cases within 3 years, despite adherence to current guideline-directed medical therapy (6, 7). Notably, only one-third of these events are related to culprit lesions at the index event, while the other two-thirds are related to non-culprit lesions (84), underscoring the importance of plaque progression. Persistent coronary inflammation, extending beyond the culprit lesion, likely contributes to this progression. Ischemic events associated with non-culprit lesions typically involve TCFA or lipid-rich plaques, features strongly correlated with heightened inflammation (7, 80). Consequently, there exists substantial evidence supporting the relationship between persistent inflammation and the risk of recurrent cardiovascular events (85). Furthermore, hs-CRP seems to be a stronger predictor than residual cholesterol risk of new cardiovascular events and death (86).

### 2.2 Acute myocardial injury

Following acute coronary obstruction, infarct size and the acute remodeling that follows thereafter (also called infarct expansion), are directly related to ischemic time and ensuing cell death; and reperfusion injury, the latter also known as ischemic-reperfusion injury

#### 2.2.1 Ischemic injury

The main determinant of ischemic injury in the setting of an MI is the time to reperfusion—the longer the time the greater the myocardial damage (87, 88). Additionally, myocardial oxygen consumption and the quality of collateral flow to the ischemic region are also important factors (89, 90). Once myocardial ischemia and necrosis ensue, an intense inflammatory response is triggered (29). It is now recognized that this inflammatory response does not merely follow ischemic injury, but modulates tissue response to it, mediating both acute tissue healing and scar formation, as well as chronic ventricular remodeling (91). This is a highly orchestrated process, in which all components of innate immunity are involved and follow each other in a predictable sequence (29). In response to myocyte necrosis and edema occurring during the first 12 h after coronary occlusion, granulocytes infiltrate the myocardial tissue, amplifying acute inflammation (92, 93). Granulocytes are then followed by monocytes, which dominate the infarct zone from days 2 to 7, clearing debris (phagocytosis) and engulfing dead cells (efferocytosis) (94, 95). This paves the way for a third wave composed of fibroblasts, which synthesize extracellular matrix and, together with neoangiogenesis, form the granulation tissue during the second week, which will then be gradually transformed into a mature scar after a couple of months (96).

Recent studies in mice have shown that mononuclear infiltration is composed of two very different subsets of monocytes (95). The first wave, occurring during days 2–3 after MI, is characterized by monocytes with very high phagocytic and proteolytic capacity, and highly proinflammatory cytokine production [TNF, IL-1, interleukin-6 (IL-6)] (95). In mice, these monocytes express high levels of the surface marker Ly-6C (Ly-6C^high^), a subset resembled in humans by monocytes expressing high levels of CD14 and low levels of CD16 (97). Once necrotic tissue and debris have been cleared by the first wave of monocytes, a second wave of distinctive monocytes arrives to promote tissue healing. From days 4 to 7, monocytes of less inflammatory potential and high reparative capacity are predominant, producing the fibrogenic mediator transforming growth factor beta (TGF-β) and the angiogenic mediator vascular endothelial growth factor (VEGF), thus promoting the formation of granulation tissue. In contrast with those from the first wave, these monocytes express low levels of Ly-6C in mice (Ly-6C^low^) (89). This fine-tuned balance of pro-inflammatory (M1) and reparative (M2) monocyte populations is key for physiologic myocardial healing. An exuberant inflammatory response leading to the predominance, or impaired transition, from the former to the latter, may be related to pathologic acute remodeling, i.e., excessive myocardial thinning, infarct expansion, and aneurysm/pseudoaneurysm formation (98).

#### 2.2.2 Reperfusion injury

It refers to all types of injury that are believed to be secondary to coronary reperfusion itself. These include myocardial stunning, ventricular arrhythmias, the *No-Reflow* phenomenon, and an entity called *lethal myocardial cell injury induced by reperfusion* (99). Among them, the *No-Reflow* phenomenon is the clinically most relevant. It is defined as inadequate myocardial perfusion in a territory subtended by a given epicardial coronary artery without angiographic evidence of mechanical vessel obstruction (100). Mechanistically, the key event is endothelial damage at the microcirculatory level, leading to microvascular obstruction. During reperfusion, massive infiltration of neutrophils and platelets occurs, with activation, adhesion, and migration of the neutrophils, releasing oxygen free radicals, proteolytic enzymes, and pro-inflammatory mediators (TNF-a, IL-1β). This causes endothelial and tissue damage, leading to focal and diffuse edema, and rupture of endothelial cells that then blocks flow. Also, there are neutrophil-platelet aggregates that plug capillaries, distal embolization from proximal lesions, and a release of vasoconstrictor agents from damaged endothelial cells (100). Percutaneous coronary angioplasty in the setting of ACS can result in rapid neutrophil recruitment to the site of mechanical trauma. The subsequent inflammatory cascade can provoke endothelial dysfunction, leukocyte-platelet aggregates in distal beds, and microvascular obstruction, leading to periprocedural MI and injury which are associated with poorer outcomes (101, 102).

Ischemic and reperfusion injury are processes affecting the myocardium affected by coronary obstruction. Interestingly, there is evidence that the acute inflammatory response to ischemic injury is not limited to the infarct zone but can extend well beyond (103–105). Hence, inflammation in the non-infarcted myocardium may emerge as a significant prognostic and therapeutic target. In an exploratory study conducted among patients post-ST elevation MI undergoing primary PCI, Bergamaschi et al. demonstrated that MRI-derived T2 mapping—a surrogate of tissue edema and thus of inflammation (106–108)—in the non-infarcted myocardium tended to increase with infarct size (109). Interestingly, the intensity of the T2 signal correlated with a higher risk of major adverse cardiac events (MACE) during follow-up, primarily driven by a heightened risk of reinfarction related to non-culprit coronary arteries. However, higher T2 values were not correlated with the presence of multivessel disease or coronary anatomical complexity. This observation might underscore the role of inflammation not only in non-culprit atheroma but also in non-culprit myocardium, as the latter may not simply mirror but actively contribute to plaque progression or instability.

### 2.3 Chronic left ventricular (LV) remodeling

Late in the course of MI, changes in LV size, shape, and thickness involving both the infarcted and non-infarcted myocardial segments are observed (26, 27). Adverse remodeling resulting in LV dilatation strongly correlates to adverse cardiovascular outcomes, such as heart failure (HF) and mortality (28, 110).

Chronic LV dilatation initiates early after MI and progresses for months or years thereafter, resulting in dilatation of the viable segments of the LV, as a compensatory mechanism for maintaining stroke volume despite the loss of significant myocardial mass (26, 27). The acute drop in stroke volume fosters the activation of a neurohumoral response, resulting in both hemodynamic and molecular changes. However, the neurohumoral response does not account for the discrepancy observed between the size of the initial MI and the magnitude of chronic LV remodeling. The inflammatory process involved in infarct healing and acute remodeling may also affect chronic LV remodeling (111, 112). In fact, the balance between a pro-inflammatory response with matrix degradation vs. a reparative response with collagen deposition may explain different degrees of late LV dilation (113, 114). Elevated levels of CRP measured at hospital admission, during the first days, or at discharge consistently predict adverse ventricular remodeling and HF (115, 116). On the other hand, persistent post-MI inflammation represents an additional potential pathogenetic mechanism of adverse ventricular remodeling. Proinflammatory cytokines such as TNF-a, IL-1β and IL-18 can exert direct actions on cardiomyocytes, leading to a cardio-depressant effect, cytotoxicity, and induction of apoptosis (114).

## 3 Colchicine: mechanisms of action

Colchicine is a botanical alkaloid derived from the flower Colchicum autumnale. Its historical use dates back to ancient Egypt, as documented on the Ebers papyrus around 1550 BC, where it was employed as a remedy to alleviate pain and swelling (117). Traditionally indicated for acute gout flares and Familial Mediterranean Fever (FMF) (118, 119), colchicine has gained recognition as a potential therapeutic option for various inflammatory conditions, including pericarditis and, more recently, atherosclerosis (120). Its notable attributes include low cost, widespread availability, and a favorable safety profile, rendering it an appealing strategy for long-term use.

**Figure 1:**
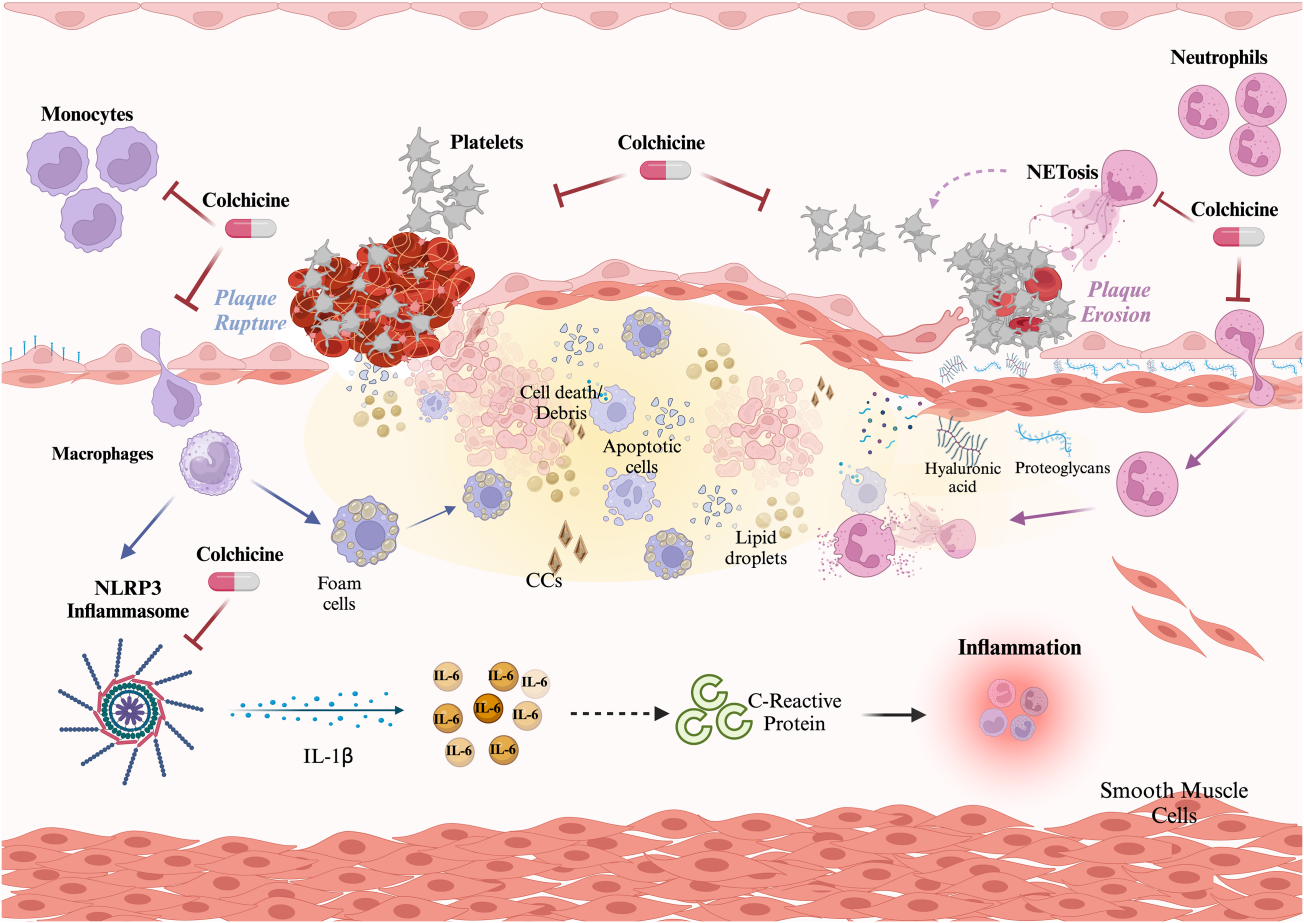
Role of colchicine in atherothrombosis. Colchicine exerts three main effects preventing atherosclerotic plaque build-up and disruption. Effect on monocytes: colchicine inhibits monocyte migration and the NLRP3 inflammasome, thus limiting the activation of the potent proinflammatory cytokine IL-1β and downstream IL-6 and C-reactive protein. Effect on neutrophils: colchicine inhibits neutrophil chemotaxis, and endothelial adhesion, and reduces deformability and motility, thus hindering recruitment and extravasation. Additionally, colchicine reduces NETosis. Both effects on neutrophils contribute to plaque stability. Effect on platelets: colchicine reduces platelet aggregation both directly and through NETs reduction. Created with Biorender.com.

The primary elucidated mechanism of action of colchicine involves the inhibition of tubulin polymerization, leading to cytoskeleton disruption and consequential impairment of pivotal cellular functions such as mitosis, intracellular transport, exocytosis, and phagocytosis (121). Nevertheless, emerging evidence suggests that colchicine may exert effects on other key aspects of the inflammatory process, potentially influencing monocytes/macrophages, neutrophils, and platelets (Figure 1).

### 3.1 Colchicine inhibition of the NLRP3 inflammasome

The NLRP3 inflammasome is a cytosolic, multimeric protein complex composed of three distinct components: NLR family pyrin domain-containing 3 (NLRP3), the adaptor protein apoptosis-associated speck-like protein containing a caspase-recruitment domain (ASC), and caspase-1 (122, 123). Triggered by various activators such as monosodium urate (MSU), cholesterol crystals, and ATP, a two-step process is initiated, leading to the activation of the potent inflammatory cytokines IL-1β and IL-18 (124–126). The NLRP3 inflammasome plays a central role in up-regulating the inflammatory milieu in ACS patients, making it an attractive target for modulating ACS-associated athero-inflammation.

Both basic and clinical studies have demonstrated the inhibitory effect of colchicine on the NLRP3 inflammasome. The initial study by Martinon and colleagues conducted on THP1 cells exposed to MSU crystals, revealed the suppressive effect of colchicine upon the NLRP3 inflammasome (127). Additionally, in bone marrow-derived macrophages and peripheral blood monocytes from FMF patients, colchicine administration markedly suppressed IL-1β expression (128). Furthermore, in peripheral blood monocytes from ACS patients, colchicine inhibited caspase-1 activity and IL-1β production (52).

While the exact pathway of colchicine's inhibition of the NLRP3 inflammasome remains elusive, recent studies have provided important insights. Using J774 macrophages treated with inflammasome inducers, Misawa et al. demonstrated that colchicine administration hindered the intracellular transport of ASC, preventing the co-localization of NLRP3, ASC, and caspase-1, thereby impeding inflammasome effector activity (129). Another study by Otani and colleagues observed the inhibitory effect of colchicine on caspase-1 protein expression in a mice model of NSAID small intestinal injury, without affecting mRNA levels of NLRP3 or IL-1 (130). Pore formation is a key step in ATP-induced inflammasome activation. Colchicine was shown to strongly inhibit P2X7 membrane pore formation in ATP-stimulated mouse peritoneal macrophages, resulting in decreased reactive oxygen species and IL-1β release (131). These findings, though diverse, collectively suggest that colchicine modulates various aspects of the NLRP3 inflammasome, particularly affecting its effector functions.

Regardless of the precise mechanism, the inhibition of monocyte/macrophage cytokine production by colchicine in ACS patients holds the potential to modulate processes following acute coronary occlusion and myocardial necrosis, as will be discussed later.

### 3.2 Colchicine effect on neutrophils

Previous research has extensively described the impact of colchicine on neutrophil function (132–134). Of importance, as a result of the lack of the P glycoprotein efflux pump, the drug accumulates in abundance within neutrophils, affecting their activity (135). In brief, colchicine impairs neutrophil chemotaxis and endothelial adhesion, seemingly through effects on E- and L-selectin. Additionally, colchicine appears to influence neutrophil deformability and motility, further hindering recruitment and extravasation.

Levels of NETosis positively correlate with myocardial infarct size, and NETs release inversely associates with myocardial perfusion after coronary angioplasty in ACS patients (136). Colchicine administration 6–24 h before coronary angioplasty in ACS patients resulted in a seven-fold lower production of NETs, measured in the coronary sinus during the procedure (137). Interestingly, this effect appeared to be driven by cytoskeleton α-tubulin stabilization, impairing NETs transport and release. Supporting these findings, colchicine has also been shown to limit NETosis in gallstones (138) and in neutrophils isolated from individuals with Behcet's disease (139).

Neutrophils and NETs actively contribute to plaque destabilization, complications, and myocardial remodeling post-MI. Therefore, their inhibition by colchicine could positively impact ACS patients.

### 3.3 Colchicine effect on platelets

Platelet activity is crucial for intracoronary thrombus formation following plaque destabilization and complications (i.e*.*, rupture or erosion) (140). NETs, on the other hand, can promote thrombosis by enhancing all platelet functions (adhesion, activation, and aggregation) and increasing the accumulation of prothrombotic factors such as von Willebrand factor and fibrinogen (141).

*In vitro*, colchicine has been shown to reduce platelet aggregation by interfering with cytoskeleton rearrangement through cofilin and LIM domain kinase 1 inhibition (142). In healthy subjects, a single oral dose of 1.8 mg has been shown to reduce leukocyte-platelet aggregation (both monocyte and neutrophil) and levels of surface markers of platelet activity, including p-selectin and PAC-1 (activated GP IIb/IIIa) (143). Moreover, in another experiment, platelets from patients receiving dual antiplatelet therapy incubated with colchicine resulted in a reduction in Thrombin Receptor Activating Peptide (TRAP)-induced platelet aggregation (144). Furthermore, in subjects with clopidogrel resistance, the addition of colchicine also inhibited ADP-induced platelet aggregation (144).

Given the role of NETs in coronary thrombosis (145), it is plausible that the inhibition of NETs formation by colchicine, as previously described, can result in reduced thrombus production and thus a more favorable phenotype after acute plaque destabilization.

## 4 Role of colchicine in acute coronary syndrome

### 4.1 Atherosclerotic plaque build-up and disruption and prevention of acute coronary events

Evidence from both animal models and clinical studies supports a positive effect of colchicine upon atherosclerotic plaque formation. In a rabbit model of atherosclerosis induced by a high cholesterol diet and balloon endothelial denudation, colchicine treatment reduced the relative increase in aortic wall volume, measured as normalized wall index, and inflammation, measured as 18F-FDG uptake in PET/CT imaging, which could potentially lead to plaque stabilization (146). Furthermore, in a prospective non-randomized observational study including 80 patients with recent ACS, colchicine administration was associated with a reduction in low attenuation plaque volume (LAPV)—a CT scan surrogate of plaque instability and predictor of future coronary events. A positive correlation between LAPV and reduced hs-CRP levels was also reported (147).

Two randomized control trials (RCT) have examined the impact of colchicine on hard clinical outcomes in the aftermath of an ACS. The COLCOT trial randomized 4,745 patients to receive colchicine (0.5 mg daily) or placebo within 30 days post-MI. Colchicine led to a significant reduction of the primary outcome (a composite of death from cardiovascular causes, resuscitated cardiac arrest, myocardial infarction, stroke, or urgent hospitalization for angina leading to coronary revascularization) by 23% (HR 0.77; 95% CI 0.61–0.96; *p* = 0.02). This was mainly driven by a significant reduction in the incidence of stroke (HR 0.26; 95% CI 0.10–0.70) and urgent hospitalization for angina leading to coronary revascularization (HR 0.50; 95% CI 0.31–0.81) (148). Interestingly, in a *post-hoc* analysis of COLCOT, time-to-treatment initiation (i.e., length of time between the index MI and the initiation of colchicine) was inversely correlated with colchicine clinical benefit. Indeed, when administered in-hospital within the first 3 days after the event, colchicine was associated with a 48% reduction in the risk of ischemic events, which contrasted with a lack of benefit when started later (4–7 days, and 7–30 days) (149). A second study, the COPS trial, was an Australian-based study that randomly assigned 795 patients diagnosed with MI or unstable angina to receive colchicine (0.5 mg BID for 1 month, then 0.5 mg daily for 11 months) vs. placebo (150). Although the original trial failed to demonstrate a benefit on the 1-year primary outcome, an extended 24-month follow-up did show a significant 40% reduction in the composite of all-cause mortality, ACS, ischemia-driven- unplanned-urgent revascularization, and non-cardioembolic ischemic stroke (151). Of note, just as in COLCOT, the main outcome was driven by a significant reduction in urgent revascularization (HR, 0.19; 95% CI 0.05–0.66; *p* = 0.009) (151). It is noteworthy that the magnitude of the benefit obtained with colchicine in patients with previous ACS is comparable to that achieved by each of the mainstay therapies for the secondary prevention of coronary artery disease—such as antiplatelet agents and statins—and has been achieved against a background of modern optimal treatment with these therapies (152, 153). Table 2 summarizes the available data on colchicine in atherosclerotic plaque stabilization and prevention of acute coronary events.

**Table 2:** Evidence for the use of colchicine on atherosclerotic plaque stabilization and prevention of acute coronary events.

Preclinical studies					
Trial	Animal model	Disease induction	Colchicine usage^a^	Main findings	Length of intervention
Cecconi et al. (146)	New Zealand White Rabbits	Balloon endothelial denudation plus high cholesterol diet	0.2 mg/kg/day 5 days/week, SQ	Reduction of the increase in aortic wall volume and inflammation	18 weeks
Clinical studies (Phase 2 and 3)					
Trial	Key inclusion criteria	No of patients	Treatment^a^	Main results	Follow up (mean)
Vaidya et al. (147)	ACS (<1 month)	80	Colchicine 0.5 mg QD plus OMT vs. OMT alone	↓ Low attenuation plaque volume in CCTA (40.9% vs. 17%) ↓ total atheroma volume	12 months
COLCOT, Tardif et al. (148)	MI (treated with PCI) within 30 days	4,745	Colchicine 0.5 mg QD plus OMT vs. OMT alone	↓ 23% MACE ↓ 84% Stroke ↓ 50% urgent hospitalization for angina leading to coronary revascularization	19.5 months
COPS, Tong, Quinn, et al. (150)	ACS treated with PCI or OMT	795	Colchicine 0.5 mg BID for 1 month, then 0.5 mg QD for 11 months vs. Placebo	↓ 84% Ischemia-driven urgent revascularization ↓ Death from any cause (8 vs. 1 patients)	12 months
COPS, Tong, Bloom, et al. (151)	ACS treated with PCI or OMT	795	Same as above, no colchicine or placebo from months 13 to 24	↓ 41% MACE ↓ 81% Ischemia-driven urgent revascularization	24 months

^a^ Oral administration unless stated otherwise. SQ, subcutaneous; ACS, acute coronary syndrome; OMT, optimal medical therapy; CCTA, coronary computed tomography angiography; MI, myocardial infarction; PCI, percutaneous coronary intervention; MACE, major adverse cardiovascular event.

### 4.2 Acute myocardial injury

**Figure 2:**
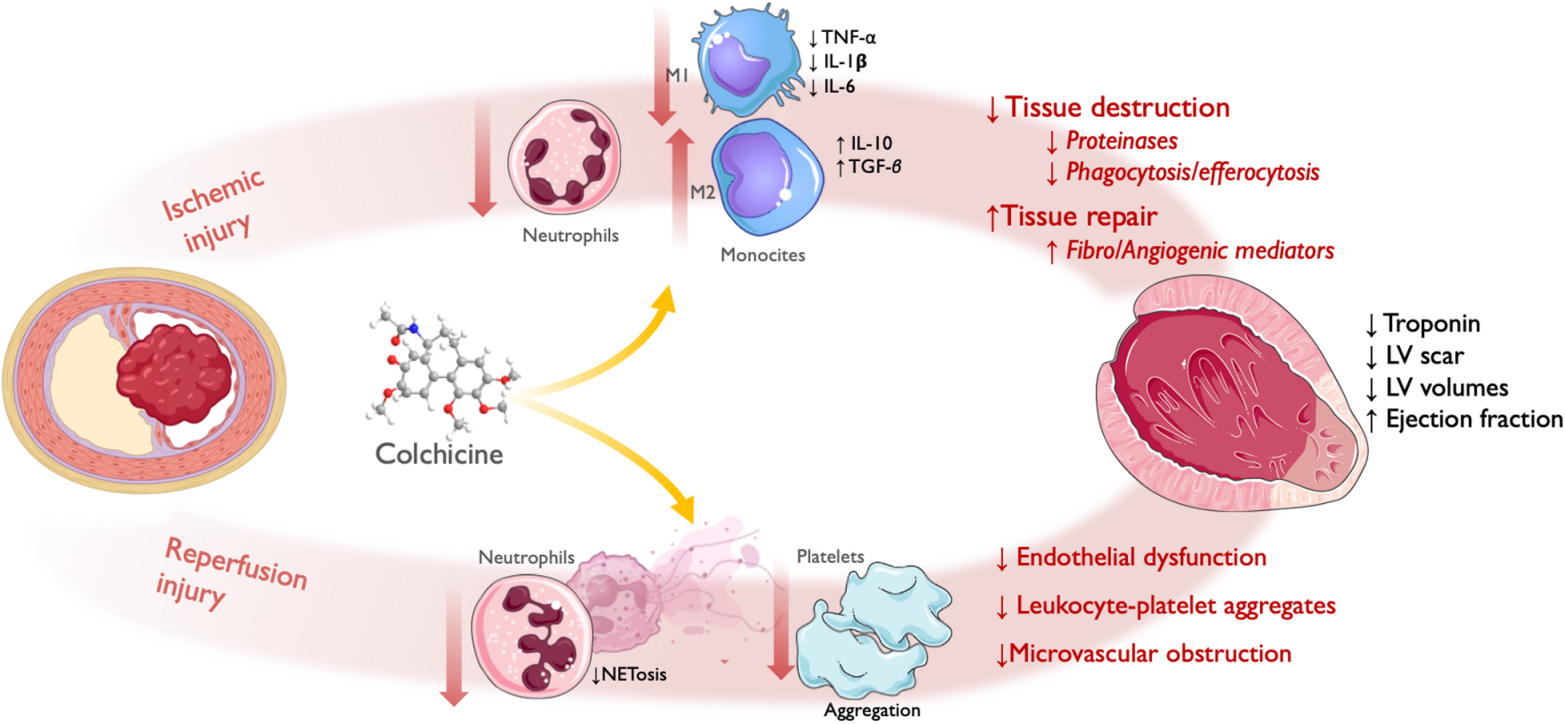
Effect of colchicine in infarct size/acute left ventricular remodeling. Following acute coronary obstruction, infarct size and acute LV remodeling are directly related to both ischemic injury and reperfusion injury (referred here as PCI-related MI). Ischemic injury: colchicine shows reduced infiltration of neutrophils and monocytes, with attenuated M1 (Pro-inflammatory)-monocyte and increased M2 (Reparative)-monocyte response. This translates into reduced myocardial tissue destruction, with less release of proteinases, and inflammatory cytokines and reduced phagocytosis/efferocytosis by activated monocytes; and enhanced tissue repair, with increased release of fibrogenic and angiogenic mediators leading to granulation tissue formation. Reperfusion injury: colchicine lowered NETosis and platelet aggregation, which translated into reduced endothelial dysfunction, leukocyte-platelet aggregates, and microvascular obstruction. Therefore, by reducing the magnitude of ischemic and reperfusion injury, colchicine administration during the acute phase of ACS has been shown to reduce infarct size, as assessed by levels of myocardial enzyme release and LV scar size; and acute LV remodeling, as assessed by LV volumes and ejection fraction. LV, left ventricle; PCI, percutaneous coronary intervention; NET, neutrophil extracellular traps; ACS, acute coronary syndrome. Icons from Servier Medical Art, licensed under CC BY 4.0.

Early colchicine administration may modulate the initial inflammatory response triggered by myocardial necrosis and the subsequent neutrophil recruitment during ischemia-reperfusion (IR) injury, impacting infarct size and acute myocardial remodeling (Figure 2).

Two animal studies explored the effect of colchicine after myocardial necrosis using mice models with permanent ligation of the left anterior descending coronary artery. In the study by Fujisue et al, colchicine administered orally after the onset of MI attenuated the inflammatory response by reducing the infiltration of inflammatory cells—both granulocytes at 24 h and macrophages at 3 and 7 days -, the activation of the NLRP3 inflammasome, and the levels of pro-inflammatory cytokines in the infarcted myocardium (154). This resulted in a reduced acute LV remodeling, showing less expansion of the LV scar size at histology and lower LV diastolic and systolic diameters, as well as higher ejection fraction at 1 week, which translated into reduced short-term mortality due to cardiac rupture (154). Likewise, in another study by Li et al., colchicine showed reduced acute cardiac remodeling as assessed by echocardiography at 7 and 28 days, and increased mice survival; interestingly, in this study the anti-inflammatory effect was mediated by a reduction in NETosis (155).

Two studies have explored the effect of colchicine in I-R injury using mice models with transient ligation of the left coronary artery. Firstly, Akodad et al. tested intraperitoneal administration of low-dose colchicine before reperfusion and showed decreased myocardial injury as assessed by troponin levels and infarct size in autopsy at 24 h. This effect was accompanied by a decrease in plasma levels of key cytokines implicated in the post-ischemic inflammatory response, such as IL-6 and Monocyte Chemoattractant Protein-1 (MCP-1) (156). Secondly, Mori et al. showed that intraperitoneal administration of colchicine starting one day after artery ligation demonstrated reduced macrophage infiltration in infarcted areas on days 3 and 7 (157). The two studies cited above showed a decrease in plasma levels of MCP-1. Interestingly, the chemokine receptor CCR2 that responds to MCP-1 predominates in the proinflammatory Ly-6C ^(high)^ monocyte subset (M1), underpinning the possible role of colchicine in modulating the balance of the main monocyte subsets implicated in post-MI inflammatory response, in favor of a more reparative Ly-6C ^(low)^ (M2) phenotype. Furthermore, in the study by Fujisue et al., colchicine tended to attenuate M1 cytokines (TNF-α, IL-1β, and IL-6) and to increase M2 cytokines [interleukin-10 (IL-10) and TGF-β], suggesting that this drug could affect the M1/M2 balance (143). The colchicine effect upon MCP-1 expression has also been confirmed in human samples from patients with ACS, impairing human-derived mononuclear cell migration capacity (158).

There is also clinical evidence of the effect of colchicine in reducing infarct size in humans. As mentioned above, colchicine administration 6–24 h before coronary angioplasty in ACS patients resulted in a seven-fold lower production of NETs, measured in the coronary sinus during the procedure (137), which may translate into reduced infarct size. However, results from phase 2 RCTs have been conflicting. In the pivotal study conducted by Deftereos et al., peri-PCI colchicine administration to STEMI patients significantly reduced CK-MB area-under-the-curve concentrations and, in a subset of patients, infarct size assessed by cardiac magnetic resonance (MRI) 1 week after MI (159). Conversely, in the CONVERT-MI, a very similar study in patients with STEMI, colchicine showed no effect on infarct size, as assessed through both biomarkers and MRI (160).

Two RCTs have evaluated the impact of colchicine in percutaneous coronary intervention (PCI) related MI. The Colchicine–PCI randomized controlled trial evaluated the effect of colchicine administration just before (1–2 h) PCI, in a mixed group of patients with stable angina and ACS. Pre-procedural colchicine did not protect against PCI-related myocardial injury (including PCI-related MI and MACE at 30 days) (161). Importantly, although they found that colchicine did decrease IL-6 and hs-CRP levels 24 h post PCI, it did not attenuate the increase of these biomarkers at 8 h, which may point to a lack of an anti-inflammatory effect of colchicine at the time of PCI, due to the short time of administration before the procedure. Thus, this might imply that the pharmacodynamic properties of colchicine require a longer time for the onset of its anti-inflammatory effect. Bearing this in mind, Cole and colleagues designed a similar RCT, administering colchicine to a mixed group of stable angina and ACS patients, but with a longer lead-in administration time before PCI (6–24 h) (162). They showed that colchicine administration significantly reduced major and minor peri-procedural MI and injury, especially in NSTEMI patients. Colchicine also significantly reduced pre-PCI inflammatory cytokine levels (IL-6, IL-1β, TNF-α, IFN-γ), and white blood cell counts, with no differences in post-PCI values (163). Table 3 summarizes the available studies on colchicine and acute myocardial injury.

**Table 3:** Evidence for the use of colchicine on acute myocardial (i.e., ischemia and reperfusion) injury in ACS.

Preclinical studies					
	Animal model	Disease induction	Colchicine usage^a^	Length of intervention	Main findings
Fujisue et al. (154)	C57BL/6J mice	Permanent ligation of left anterior descending coronary artery	0.1 mg/kg	7 days after MI	- Reduced infiltration of granulocytes and monocytes - Attenuation of pro-inflammatory - Cytokines and NLRP3 inflammasome - Reduced acute LV remodeling: reduced scar size, lower diastolic and systolic volumes, higher EF at day 7
Li et al. (155)	C57BL/6J mice	Permanent ligation of left anterior descending coronary artery	0.1 mg/kg	7 or 28 days after MI	- Inhibition of NETs formation - Reduced acute LV remodeling with improved EF at day 7
Akodad et al. (156)	C57BL/6J mice	Ligation of left coronary artery followed by reperfusion	0.4 mg/kg, 1 or 2 mg/kg IP	25 min before reperfusion	- Reduced IL-6 and MCP-1 - Reduced infarct size and circulating T troponin at 24 h after ischemia-reperfusion
Mori et al. (157)	Wistar male rats	Ligation of left coronary artery followed by reperfusion	0.4 mg/kg IP	3 or 7 days after reperfusion	- Reduced macrophage infiltration in the infarcted area - Decrease in MCP-1
Clinical studies					
Trials	Key inclusion criteria	No of patients	Treatment^a^	Main results	Follow up (mean)
Deftereos et al. (159)	STEMI <12 h treated with PCI	151	Colchicine loading dose after diagnostic angiography, followed by 0.5 mg BID vs. placebo	↓ CK-MB and troponin ↓ Infarct size (MRI)	5 days
COVERT MI, Mewton et al. (160)	STEMI <12 h treated with PCI	192	2 mg loading dose, followed by 0.5 mg BID for 5 days vs. placebo	No difference in infarct size, CK levels, or inflammatory markers	3 months
COLCHICINE—PCI, Shah et al. (161)	CCS or ACS referred to PCI	400	Colchicine 1.8 mg pre-PCI vs. placebo	No difference in risk of PCI-related MI or injury, or MACE at 30 days	30 days
COPE –PCI, Cole et al. (162)	CCS or NSTEMI going to PCI	75	Colchicine 1.5 mg pre-procedural (6–24 h before angiography) vs. placebo	↓ 41% PCI-related MI and injury ↓ total WBC	24 h
COPE—PCI, Cole et al. (163)	CCS or NSTEMI going to PCI	75	Same as above	↓ levels of IL-1β, IL-6, IL-10, TNF*α* and WBC ↓ PCI-related MI and injury	24 h

^a^ Oral administration unless stated otherwise; MI, myocardial infarction; NLRP3, nucleotide-binding oligomerization domain-like receptor, pyrin domain-containing; LV, left ventricle; EF, ejection fraction; NET, neutrophil extracellular traps; IP, intraperitoneal; IL-6, interleukin 6; MCP-1, monocyte chemoattractant protein-1; STEMI, ST-elevation myocardial infarction; PCI, percutaneous coronary intervention; CK, creatine kinase; CK-MB, creatin kinase-myocardial band; MRI, magnetic resonance imaging; CCS, chronic coronary syndrome; ACS, acute coronary syndrome; NSTEMI, non-ST-elevation myocardial infarction; MACE, major adverse cardiovascular event; OMT, optimal medical therapy; WBC, white blood cells; IL-1 β, interleukin 1β; IL-10, interleukin 10; TNFα, tumor necrosis factor α.

### 4.3 Chronic left ventricular remodeling

Evidence for the role of colchicine in chronic LV remodeling is scarce, although the same preclinical animal models of MI or ischemia-reperfusion (I-R) injury can provide some insights into a potential role in late remodeling. In the aforementioned study by Fujisue et al., the reduction in post-MI acute remodeling was sustained throughout the study follow-up, with lower LV diastolic and systolic diameters, as well as higher ejection fraction at 4 weeks (154). In the model of I-R by Akodad et al., in addition to the acute cardioprotective effect, colchicine increased cardiac output in cardiac ultrasounds performed 8 weeks after transient artery ligation and lowered the histological assessment of fibrosis at 10 weeks after I-R injury (156). In the model of I-R by Mori et al, in addition to reduced macrophage infiltration, colchicine demonstrated reduced LV volumes and higher ejection fraction, as assessed by 99mTc-MIBI gated SPECT, with differences starting as early as 2 weeks after MI, but becoming statistically relevant at 8 weeks (157). Table 4 summarizes the available studies on colchicine and chronic LV remodeling. Translation of these results to clinical data is largely awaited.

**Table 4:** Evidence of colchicine in chronic left ventricular remodeling.

Preclinical studies					
	Animal model	Disease induction	Colchicine usage^a^	Length of intervention	Main findings
Fujisue et al. (154)	C57BL/6J mice	Permanent ligation of left anterior descending coronary artery	0.1 mg/kg	7 days after MI	Reduced LV diastolic and systolic diameters, as well as higher ejection fraction on echocardiography at 4 weeks Lower LV end-diastolic pressure on cardiac catheterization at 4 weeks
Li et al. (155)	C57BL/6J mice	Permanent ligation of left anterior descending coronary artery	0.1 mg/kg	7 or 28 days after MI	Improved LVEF on echocardiography at day 28 (26.2% vs. 14.8%)
Akodad et al. (156)	C57BL/6J mice	Ligation of left coronary artery followed by reperfusion	0.4 mg/kg, 1 or 2 mg/kg IP	25 min before reperfusion	Improved hemodynamic parameters (ITV) on echocardiography at 8 weeks, without differences in remodeling parameters
Mori et al. (157)	Wistar male rats	Ligation of left coronary artery followed by reperfusion	0.4 mg/kg IP	3 or 7 days after reperfusion	Reduced LV diastolic and systolic volumes and improved LVEF (42.2 vs. 28.4%) on SPECT at 8 weeks

^a^ Oral administration unless stated otherwise; MI, myocardial infarction; LV, left ventricle; EF, ejection fraction; IP, intraperitoneal; ITV, integral time velocity; SPECT, single-photon emission computed tomography.

## 5 Other anti-inflammatory therapies

During the last decades, multiple therapies that specifically target inflammation in atherosclerosis have been tested. However, thus far only agents targeting the IL-1β—IL-6 pathway have shown some efficacy in atherosclerosis. These agents include mainly the NLRP3 inhibitors and direct IL-1 and IL-6 inhibitors. Here, we briefly describe the most important recent advancements in alternative anti-inflammatory approaches for patients with ACS.

### 5.1 IL-1β inhibitors

Experimental evidence links IL-1 to atherosclerotic plaque formation, impaired vasodilation, and increased atherothrombosis (164). Interleukin-1 has two isoforms, IL-1α and IL-1β. While IL-1α ignites inflammation during MI, IL-1β expression during the subacute phase contributes to apoptosis and cardiac remodeling. As previously mentioned, in the CANTOS trial, patients with a history of MI and high levels of hs-CRP were randomized to receive either canakinumab or placebo, resulting in a 15% reduction in CV events, independent of aggressive cholesterol control (20).

Anakinra is an interleukin-1 receptor antagonist (IL-1Ra), resulting in the inhibition of both IL-1α and IL-1β. Patients with STEMI receiving anakinra showed lower levels of hs-CRP (165). Furthermore, a pooled analysis of the VCUART trials found anakinra to reduce the incidence of new-onset HF or hospitalization for HF at 1 year following STEMI (166). Although initial results seem promising, future trials testing the role of anakinra in ACS, and including a larger number of patients, are expected.

### 5.2 IL-6 inhibitors

IL-6 is an effector cytokine downstream of IL-1β, playing a significant role in atherosclerosis. Cardiomyocytes produce IL-6 under ischemia, which leads to inflammation and cytotoxicity. Tocilizumab, a monoclonal antibody blocking IL-6 signaling, has shown success in treating conditions like rheumatoid arthritis (167). In a phase II trial involving non-STEMI patients, tocilizumab was shown to significantly reduce CRP levels (168). Recently, the ASSAIL-MI trial investigated the effect of a single dose of tocilizumab on STEMI patients within 6 h of symptoms, revealing a significant improvement in MRI-derived myocardial salvage index and less microvascular obstruction. In this trial, however, the reduction in final infarct size at 6 months was not statistically different, and the beneficial effect of tocilizumab seemed to be limited to patients presenting with more than 3 h after symptom onset (22). The ongoing ARTEMIS trial is testing the effect of Ziltivekimab, a novel IL-6 inhibitor, in patients admitted with myocardial infarction. The results are eagerly anticipated (clinicaltrials.gov/NCT06118281).

### 5.3 Methotrexate

Methotrexate is an immunosuppressant drug with adenosine-mediated anti-inflammatory effects (169). It is commonly used as a treatment for systemic inflammatory diseases, including rheumatoid arthritis, psoriatic arthritis, and juvenile idiopathic arthritis. The CIRT trial administered low-dose methotrexate to patients with metabolic syndrome or type 2 diabetes with recent ACS. Methotrexate did not reduce levels of IL-1β, IL-6, or hs-CRP and was not associated with a reduction in cardiovascular events (21).

## 6 Future perspectives

As depicted in the current review, a substantial body of evidence has accrued over the past decade concerning the potential beneficial impact of colchicine on ACS patients. Animal models indicate effects extending beyond tubulin polymerization inhibition, encompassing the modulation of monocyte, neutrophil, and platelet activity—key cell types implicated in ACS pathophysiology.

Colchicine may have a different impact on each plaque phenotype. Its demonstrated inhibitory effect on the NLRP3 inflammasome, with inhibition of monocyte/macrophage cytokine production, may translate into reduced lipidic/necrotic core volume and increased fibrous cap thickness, thus reducing the probability of plaque rupture. As previously mentioned, this has been suggested by one observational study, in which colchicine administration was associated with a reduction in low attenuation plaque volume measured by CT scan (147), as well as in an animal model of atherosclerosis (128). The COCOMO-ACS is an RCT that will provide more evidence on the effects of colchicine upon high-risk features of coronary plaques, as assessed by OCT (170). Moreover, given its crucial effects on neutrophils and NETosis, it is plausible that colchicine might have also an important effect on the thrombus formation during plaque erosion.

**Figure 3:**
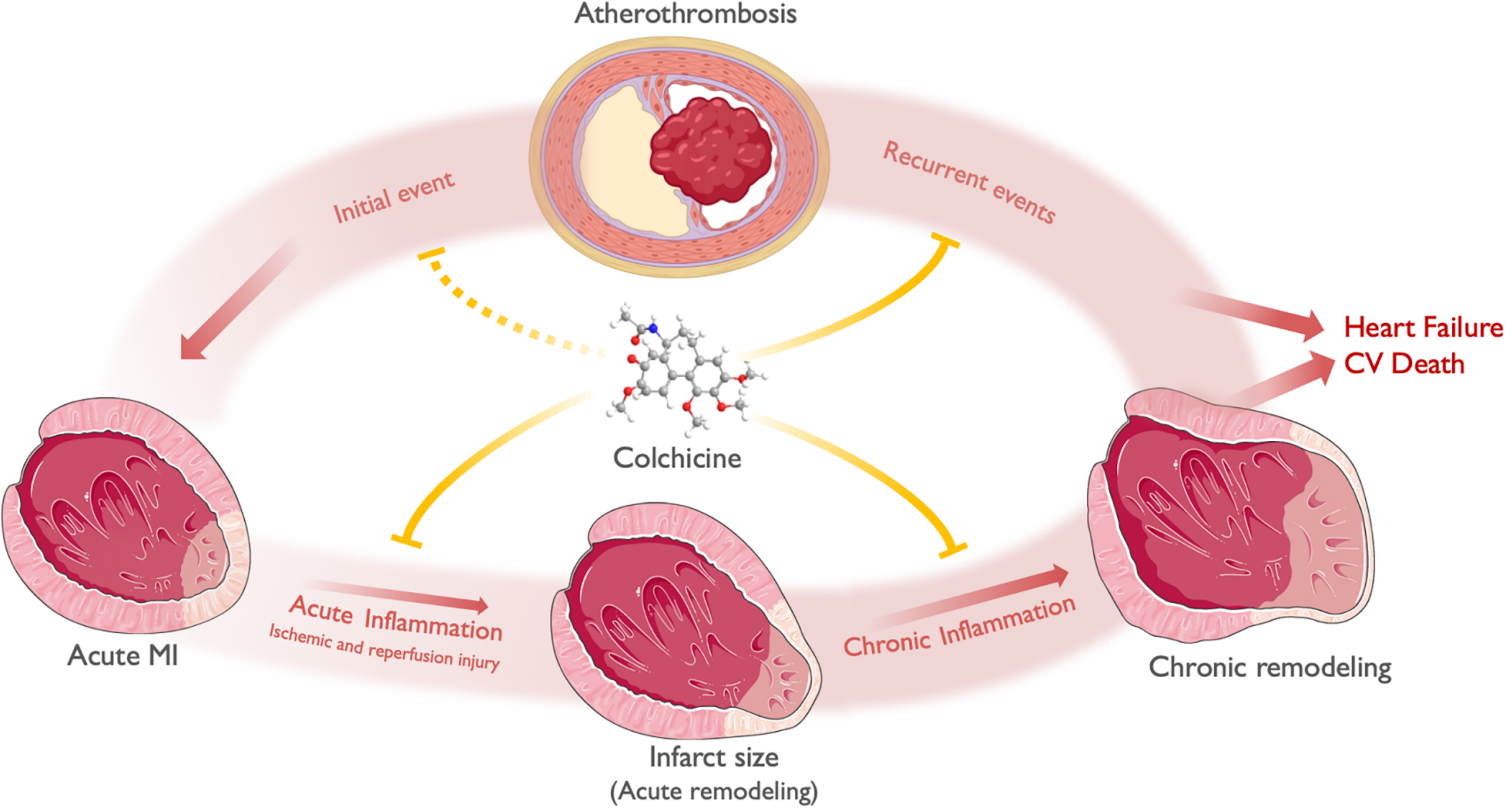
A general framework of the effect of colchicine in ACS. ACS most commonly derives from atherosclerotic plaque disruption (Initial event), leading to acute ischemia and MI. Acute inflammation along with time to reperfusion and other hemodynamic factors, mediates ischemic and reperfusion injury and determines infarct size and acute LV remodeling. Chronic inflammation, alongside neurohumoral activation, determines chronic LV remodeling. Both chronic LV remodeling and recurrent ischemic events determine the progression to heart failure and cardiovascular death. Colchicine exerts its anti-inflammatory effects both in the myocardium and the atherosclerotic plaque. At the myocardial level, colchicine attenuates acute inflammation -both ischemic and reperfusion injury-, thus limiting infarct size and acute remodeling. Subsequently, colchicine attenuates chronic low-grade inflammation, limiting chronic LV remodeling (i.e., chamber dilation and reduced EF). At the atheroma level, colchicine reduces plaque instability features, thus preventing plaque disruption and recurrent events. Theoretically, it may also prevent the occurrence of an initial ACS in patients with chronic coronary syndromes. Finally, the effects of colchicine in both attenuating acute and chronic LV remodeling after an ACS, in addition to the prevention of recurrent ACS, may translate into reduced heart failure and CV death. Icons from Servier Medical Art, licensed under CC BY 4.0.

This postulated effect of colchicine in plaque stabilization has been evidenced by randomized clinical trials demonstrating its efficacy in reducing new ischemic events. Additionally, colchicine shows potential in reducing infarct size and cardiac remodeling following an ACS. Lastly, its effects on NETs are expected to mitigate ischemia/reperfusion injury, leading to improved reperfusion during percutaneous coronary intervention (PCI). Figure 3 depicts a proposed general framework of the effect of colchicine in ACS.

The 2021 ESC guidelines on the prevention of cardiovascular disease endorse colchicine as an alternative for the secondary prevention of cardiovascular disease, particularly for individuals experiencing recurrent events despite optimal medical therapy (class IIb recommendation, level of evidence A) (171). Moreover, colchicine has received approval from the FDA and Health Canada for the prevention of cardiovascular events in patients with established atherosclerotic disease or multiple cardiovascular risk factors (172).

Nevertheless, several other aspects need to be addressed before the widespread use of this drug in patients with ACS. Firstly, the timing of initiation remains unclear. A substudy of COLCOT indicated that the benefit of colchicine was limited to those patients who commenced the drug within the first 3 days following the event (150). Moreover, if an effect on reperfusion and acute remodeling is desired, early initiation of colchicine upon admission and before percutaneous coronary intervention (PCI) may be necessary. Secondly, targeting higher-risk populations (e.g., those with increased inflammatory residual risk) could potentially enhance the drug's efficacy. While hs-CRP is a recognized biomarker that may be reduced by colchicine treatment (173), large clinical trials did not select patients based on its levels. The ongoing COLCARDIO-ACS study, which aims to randomize 3,000 post-MI patients with persistently elevated hs-CRP to receive either colchicine or placebo, may provide valuable insights into this topic. Thirdly, the current treatment for post-MI patients typically involves dual antiplatelet therapy, lipid-lowering treatment with one or two drugs, beta-blockers, angiotensin inhibitors, and possibly an SLGT-2 inhibitor. This regimen can be further tailored based on MI severity and patient comorbidities. Therefore, how to integrate a new drug into this already comprehensive treatment approach remains to be defined. In this context, the two aspects previously discussed—timing of initiation and tailoring—require clarification to provide recommendations on how best to utilize colchicine in these patients.

Finally, while data on the long-term effects of colchicine in ACS patients is promising, the emergence of increased non-cardiovascular deaths among patients randomized to colchicine (174) warrants caution and needs to be addressed in future trials. Several ongoing trials seek to elucidate many of these areas of uncertainty (Table 5).

**Table 5:** Summary of ongoing studies of colchicine in acute coronary syndrome.

Study	Design	Target population	Primary outcome	Follow-up	Country
Colchicine and spironolactone in patients with mi/synergy stent registry (CLEAR SYNERGY) NCT03048825	Phase 3, prospective, randomized, placebo-controlled	STEMI and NSTEMI (*N* = 7,000)	MACE	1 year	USA
Colchicine effects on cardiovascular outcomes in acute coronary syndrome study (COLCARDIO-ACS) ACTRN 12616000400460	Phase 3, prospective, randomized, placebo-controlled	ACS 4–52 weeks after event + elevated hs-CRP (*N* = 3,000)	MACE	3 years	Australia
Effects of colchicine in patients with myocardial infarction NCT 04218786	Phase 2, prospective, randomized, double-blind	ACS (*N* = 800)	MACE	3 months	Pakistan
Colchicine for reduction of periprocedural myocardial injury in percutaneous coronary intervention NCT05745818	Prospective, open-label, randomized cohort study	ACS and stable patients undergoing elective PCI (*N* = 300)	MACE	1 month	Egypt
Colchicine in patients Undergoing Coronary Artery Bypass Grafting After Acute Coronary Syndrome (COCAR) NCT05726019	Prospective, open-label, randomized study	ACS, with indication for myocardial revascularization surgery (*N* = 100)	Composite of post-pericardiotomy syndrome, postoperative fibrillation, and periprocedural myocardial infarction	1 month	Brazil
Effect of colchicine on MMP-9, NOX2, and TGF-β1 in myocardial infarct NCT05709509	Prospective, randomized, parallel assignment (medical treatment vs. revascularization with or without colchicine)	Late presentation STEMI (>12 h) (*N* = 148)	Left ventricular end-diastolic volume and relevant biomarkers	1 month	Indonesia
Effect of colchicine on coronary reperfusion in patients with acute coronary syndrome NCT05472337	Prospective, randomized, colchicine vs. no colchicine	ACS patients undergoing PCI (*N* = 50)	Index of microvascular resistance pre-post PCI, MRI infarct size	6 weeks	Chile
Short course low dose oral colchicine after ST elevation myocardial infarction (STEMI) NCT06020300	Prospective, randomized, colchicine vs. placebo	STEMI (*N* = 64)	Serum troponin I change from arrival to discharge And MACE	1 month	Malaysia

MI, myocardial infarction; STEMI, ST-elevation myocardial infarction; NSTEMI, non-ST-elevation myocardial infarction; MACE, major adverse cardiovascular event; hs-CRP, high sensitivity-C reactive protein; ACS, acute coronary syndrome; PCI, percutaneous coronary intervention; MRI, magnetic resonance imaging.

## 7 Conclusion

The pathophysiological mechanisms underlying an ACS consist of coronary plaque destabilization and *in situ* thrombosis, acute myocardial injury, and chronic left ventricular remodeling. Through the modulation of monocyte/macrophage, neutrophil, and platelet activity, colchicine holds promise for positively influencing patients with ACS. This may lead to a reduced rate of subsequent ischemic events, smaller MI, and a more favorable remodeling. Although recent evidence supports its use in post-MI patients, further research is warranted to determine the optimal context for utilizing this resurging therapeutic agent.
